# Behavioural and neural responses of crabs show evidence for selective attention in predator avoidance

**DOI:** 10.1038/s41598-022-14113-0

**Published:** 2022-06-15

**Authors:** Zahra M. Bagheri, Callum G. Donohue, Julian C. Partridge, Jan M. Hemmi

**Affiliations:** 1 School of Biological Sciences, The University of Western Australia, Perth, Australia; 2 The UWA Oceans Institute, The University of Western Australia, Perth, Australia; 3 Harry Butler Institute, Murdoch University, Perth, WA Australia

**Keywords:** Behavioural ecology, Decision, Sensory processing

## Abstract

Selective attention, the ability to focus on a specific stimulus and suppress distractions, plays a fundamental role for animals in many contexts, such as mating, feeding, and predation. Within natural environments, animals are often confronted with multiple stimuli of potential importance. Such a situation significantly complicates the decision-making process and imposes conflicting information on neural systems. In the context of predation, selectively attending to one of multiple threats is one possible solution. However, how animals make such escape decisions is rarely studied. A previous field study on the fiddler crab, *Gelasimus dampieri*, provided evidence of selective attention in the context of escape decisions. To identify the underlying mechanisms that guide their escape decisions, we measured the crabs’ behavioural and neural responses to either a single, or two simultaneously approaching looming stimuli. The two stimuli were either identical or differed in contrast to represent different levels of threat certainty. Although our behavioural data provides some evidence that crabs perceive signals from both stimuli, we show that both the crabs and their looming-sensitive neurons almost exclusively respond to only one of two simultaneous threats. The crabs’ body orientation played an important role in their decision about which stimulus to run away from. When faced with two stimuli of differing contrasts, both neurons and crabs were much more likely to respond to the stimulus with the higher contrast. Our data provides evidence that the crabs’ looming-sensitive neurons play an important part in the mechanism that drives their selective attention in the context of predation. Our results support previous suggestions that the crabs’ escape direction is calculated downstream of their looming-sensitive neurons by means of a population vector of the looming sensitive neuronal ensemble.

## Introduction

Survival of prey animals heavily relies on their ability to evaluate a constant stream of sensory information from often complex environments and situations. The process of making the correct decision is further complicated for animals that have limited sensory and neural processing capabilities. It is more difficult for such neural systems to separate relevant information from noise, regardless of whether it is sensory noise or the presence of irrelevant objects or movements within their environment^1,2^. For instance, prey animals often need to detect predators against highly cluttered backgrounds where distractors, such as conspicuous objects, falling leaves, or non-threatening animals, may appear as potential threats. Animals therefore need to be able to select the most relevant stimuli and ignore the rest. Such selectivity arises through attentional mechanisms, which are defined as the capacity to acquire, filter, and process only a limited subset of the available sensory input^3–5^.

Attention-like mechanisms play an important role in escape decisions. Being simultaneously approached by multiple predators enhances the level of risk^6–8^ and imposes conflicting information on the neural system. How animals deal with these challenges, what escape strategies they use, and how attentional mechanisms feed into their decisions is poorly understood. Two main attentional mechanisms have been suggested for animals to deal with multiple predators. Firstly, prey may adjust their response behaviour to minimise the combined risk from all identified threats. For instance, the lacertid wall lizard *Podarcis lilfordi* was found to escape in a direction that maximises the distance from two predators^9^. While this strategy is ideal, it requires a ‘divided attention’ mechanism and an ability to find an integrated solution. However, attention is a limited resource, and such a strategy requires a significant investment in neural processing capacity. Secondly, when presented by multiple potential predators, prey animals such as wood frog tadpoles (*Rana sylvatica*)^10^ and hemipteran water striders (*Aquarius remigis*)^11^ identify the predator that poses the highest risk and respond only to that predator. This strategy is not ideal as the less threatening predator may ultimately be successful in capturing the prey. This strategy requires ‘selective attention’, a mechanism which leads to one stimulus being preferred over alternatives that are otherwise perceived equally well when presented alone^12^. Using this mechanism prey suppresses the response to the less threatening predator and therefore reduces the processing requirements of the nervous system^13–16^. Theoretically, an alternative mechanism is to integrate the information from both predators and respond to the average signal; a strategy that does not require any attentional mechanism. It might work well in some situations, for instance when two predators approach from the same side of the animal, but it will not work when predators approach from opposite directions.

Fiddler crabs provide an excellent opportunity to investigate the role of visual attention in predator avoidance, particularly for understanding how species with relatively simple neural systems and limited sensory capabilities respond to multiple predators. Fiddler crabs rely solely on visual information to escape their avian predators and the capabilities and limitations of their visual system are well documented (reviewed by^17,18^). These attributes allow detailed interpretation of crab behaviour. Their compound eyes are situated on long stalks which are kept perpendicular to the visual horizon at all times^19,20^. Each compound eye of a fiddler crab has a 360° visual field, with a sampling resolution that varies along the visual horizon. Their small body size limits their spatial resolving power^21–23^, which peaks at 1.9 c/deg in the crabs’ lateral field of view, directly aligned with their longitudinal body orientation^24,25^. Consequently, fiddler crabs cannot resolve much spatial detail that may assist with identification of predators through shape and patterns^20,24–26^. Therefore, these crabs treat all moving stimuli above the horizon as potential threats^26,27^ and even simple dummy predators can provoke reliable anti-predator responses similar to those produced by real predators (e.g.^26,28–31^). Studies from both lab and field experiments^20,26,28,31–34^ have shown that fiddler crabs base the time and probability of their escape responses on visual cues such as expansion speed, retinal speed, angular size, contrast, and elevation above the horizon to evaluate risk^28,32,33,35^.

We previously examined the escape response of fiddler crabs under naturalistic conditions in the field, when they faced two simultaneously approaching dummy predators. The experiment was arranged such, that the predator that initially caused less retinal movement, and therefore appeared as less threatening, ultimately approached closer and became the more threatening object ^14^. The results suggested that the crabs behaved as if they only faced one predator. They responded to the predator that was, during the course of the approach, going to become the more dangerous predator and suppressed their usually earlier, response to the ultimately less threatening stimulus. These results suggested a possible role of predictive selective attention in the crabs’ escape behaviour.

Although field observations test the animals in a natural context, the natural environment is complex, highly dynamic, and often contains conflicting and novel stimuli. Consequently, accurately controlling the sensory information available to animals and identifying what information is behaviourally relevant in a functional context is difficult and often impossible. Identifying the underlying mechanisms that guide selective attention therefore also requires laboratory experiments in more controlled conditions.

Clear correlations between attentional behaviours and neural activities suggest that studying neural systems could provide details on the mechanism of selective attention that underlie animal behaviour. For instance, when honeybees (*Apis mellifera*) were presented with competing visual stimuli, the selection of the most salient stimulus by neurones in the medulla preceded ultimate behavioural choices made by the bees^36^. Tang and Juusola^37^ have shown that when flies (*Drosophila melanogaster*) faced competing motion stimuli on their left and right, they chose one stimulus at a time and attempt to rotate toward its direction. The neural output of the optic lobes in flies alternates and increases on the side chosen for body rotation and is suppressed on the opposite side. Understanding the neural mechanisms could ultimately also inform the design of novel information processing units suited to autonomous robots (e.g. ^38–40^).

The neuronal structure likely to underlie crab escape responses has been previously identified using in vivo intracellular recording. Medan et al.^41^ described four classes of Lobula Giant (LG) neurons with different sensitivities to different motion parameters. Two classes of LG neurons, “monostratified lobula giant 1” (MLG1) and “monostratified lobula giant 2” (MLG2), have been shown to be highly sensitive to looming stimuli and play a key role in the visuomotor coordination required in escape responses^41–43^. MLG1 neurons form a group of 14–16 units distributed across the lobula retinotopic mosaic^44^. Each unit has a large (> 90 deg) and non-uniform receptive field that responds to a specific part of visual space. This arrangement makes them suited to encode and convey information on the positions of objects^41,42,45,46^. In contrast, there seems to be only one element of the MLG2 neuron per lobula with a more uniform receptive field that encompasses the entire visual space of the crab (360°)^41,42^. MLG2s convey the information to adjust the speed of the escape run^43,46^. While there are no measurements of MLG neuron activity in behaving crabs, Sztarker and Tomsic^47^ have shown that changes in the response rate of MLG neurons closely reflect behavioural changes in response to changing stimulus features, viewing conditions, seasonal variation^47^ and learning^48^. In addition, Tomsic et al.^48^ have shown that the responses of MLG neurons precede the behavioural responses by 120 ms. Given this correspondence LG neurons are thought to be fundamental in the decision-making process for visually guided escape behaviours^47^. Therefore, there is clear value in targeting MLG neurons to study the mechanism of selective attention in fiddler crabs escape behaviour.

In the current study we conduct behavioural and neuronal experiments in controlled experimental conditions to test for a role of selective attention in the escape decisions of fiddler crabs. We build on previous field work by attempting to explain the strategy of stimulus selection when the crabs are exposed to multiple stimuli and to explore the mechanism of attention in this species through electrophysiology. For these purposes, we change contrast to manipulate the threat certainty level; a higher contrast is assumed to indicate a higher risk. We first measured the crabs’ behavioural and neural (i.e. MLG neuronal) responses to two identical stimuli approaching simultaneously to show that crabs selectively respond to only one and suppress the response to the other stimulus. We then tested the crabs’ responses to two simultaneous stimuli of different contrasts, intended to represent different levels of threat certainty. We compared these responses to single looming stimuli of equivalent contrasts to show that when the predation risk varies between the stimuli, crabs choose to respond only to the predator that poses the higher risk. Due to variation of spatial sampling resolution across the visual field of crabs^24,25^ and the fact that fiddler crabs run quickest when they move sideways^49^ we also analysed the effect of the crabs’ body orientation on determining the predator they ‘chose’ to run from when they face simultaneous identical stimuli.

## Material and methods

### Animals

Fiddler crabs of the species *Gelasimus dampieri* (formerly *Uca dampieri*) were collected from intertidal mudflats near Broome (− 17.9° S, 122.2°E), Western Australia. The fiddler crabs were housed in an artificial mudflat at the University of Western Australia and exposed to a tidal cycle of seawater inundation. The crabs were maintained under a daily 12L:12D light regime that includes UV and their diet was supplemented with fish food. In total 50 crabs (25 females, 25 males) and 23 crabs (14 females, 9 males) were used in the behavioural and intracellular experiments respectively. Crabs ranged from 15.2 to 23.6 mm in carapace width (measured between lateral carapace spines). To easily identify each crab, animals were transferred to a partitioned plastic tank filled to a minimum of 1 cm depth with seawater to be used for the experiments. Tidal inundation, light cycle, diet, water quality and animal care routine were the same in both facilities. Animals were approved for use under the UWA animal ethics protocol RA/3/100/1515.

### Behaviour

#### Apparatus and experimental procedure

**Figure 1:**
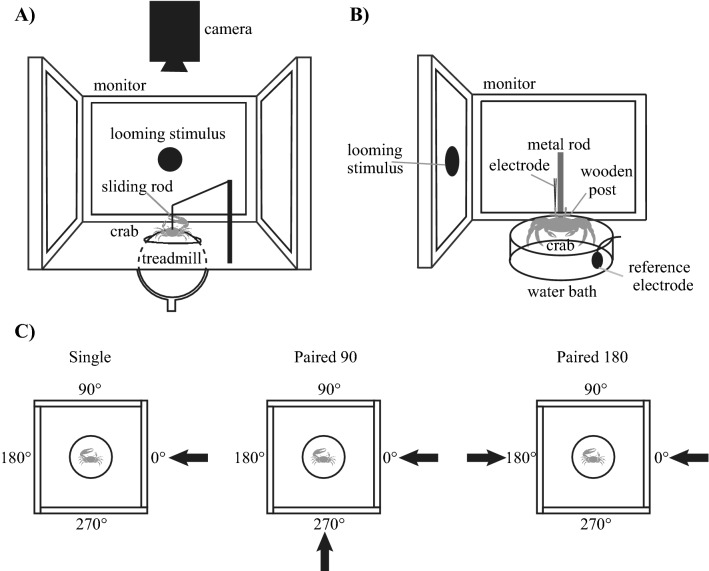
Experimental setups and stimulus configurations. (**A**) The behavioural experiment was conducted on an air cushioned treadmill. Stimuli were presented either on one or two of the four computer monitors surrounding the treadmill. The crab was held in place by a rod attached to its carapace that allowed it to slide up and down and rotate freely around the centre of the ball, but not move away. (**B**) Intracellular in vivo electrophysiology involved inserting an electrode into the optic lobe of crab to record single-neuron responses to stimuli presented on two computer monitors placed at right angles next to each other. The crab was firmly held by gluing it to a metal rod and the eyestalks were stabilized in an approximately natural position, by gluing to a wooden post. A small piece of cuticle was removed from the top of the eyestalk to provide access to the optic lobes. (**C**) The stimuli for the behavioural experiments consisted of (1) a single looming stimulus, presented on any one of the four monitors (Single). (2) A pair of looming stimuli presented on any of two adjacent monitors (Paired 90). (3) A pair of looming stimuli presented on any two opposed monitors (Paired 180). The stimuli used for electrophysiology were the same but excluded the Paired 180 configuration and stimuli were only presented on two monitors.

A treadmill setup was used to test the response of fiddler crabs to single versus paired stimuli^35^ (Fig. 1A). Two independent behavioural experiments were conducted (see Stimuli and treatment section for details) on two near identical setups that differed in ball size and weight. The treadmill for experiment 1 was an air-cushioned polystyrene foam ball (outer diameter $\approx$ 11.8 cm, weight = 8.2 g). The treadmill for experiment 2 contained a polystyrene foam ball of the same outer dimensions but was hollowed to reduce its moment of inertia (inner diameter $\approx$ 9.8 cm, weight = 5.87 g)^50^. In both cases, crabs were placed on top of the ball and tethered to a sliding, circular carbon-fibre rod by means of a small and lightweight magnetic ball (weight = 0.315 g (magnet + rod), magnet diameter = 2 mm) attached to its end. A corresponding small, magnetic disk (weight = 0.02 g, diameter = 2 mm) was glued (Loctite Superglue, ethyl cyanoacrylate) to the frontal region of the crab’s carapace. The circular cross-section of the sliding rod allowed the tethered crabs to rotate around their vertical axis and walk freely in any direction, causing the ball to rotate beneath it.

The treadmills were both surrounded by four LCD monitors (55.6 cm × 39.85 cm, 1920 × 1200 pixels at 60 Hz, Dell U2412M), positioned 27 cm away from the centre of the ball, to present the stimuli. Monitors were calibrated for linearity in contrast. Monitor radiance was measured using a radiometer (ILT1700, International Light Technologies) at all pixel values from 0 (“black”) to 255 (“white”). A look-up table was then calculated to ensure that the screens radiation changed linearly with changes in the pixel value. All visual stimuli were generated with a single PC, using a custom-built presentation and data acquisition suite based on MATLAB R2017b (MathWorks) and Psychtoolbox (Psychtoolbox-3, http://psychtoolbox.org/).

Crab behaviour was captured with a video camcorder (HDR-CX550, Sony) recording at 25 fps for experiment 1 and a digital HD video camera (FLIR Grasshopper 3) recording at 90 fps for experiment 2. In both cases, the cameras were located directly above the test arena. To avoid habituation^51,52^, each individual crab was exposed to only two stimuli on any given day and only tested every second day. Crabs were introduced into the treadmill at least three minutes before the first stimulus and their behaviour was monitored to ensure they had acclimatized before testing. The two stimuli were separated in time by a minimum of three minutes. Video sequences were synchronised to stimulus events by displaying a small square on the stimulus monitor, which only appeared when the stimulus started and disappeared when it ended. A small mirror was placed in the experimental arena to reflect the small square into the field of view of the camera and to obscure it from the crab’s field of view.

#### Stimuli and treatments

The monitors were used to display computer-generated stimuli, which simulated predators approaching the crabs on a direct collision course. Stimuli were dark, looming disks presented on a 50% grey background. All stimuli were calculated to mimic an object of 30 mm in diameter approaching with a constant velocity of 200 mm/s from a starting distance of 5000 mm. To crabs in the apparatus these stimuli thus expanded from an object with a diameter subtending 0.34° until it filled the monitor. Since fiddler crabs align their eyes to the visual horizon at all times^19,20^, a stationary grey horizontal bar was presented at the bottom of all four monitors at the level of the crab’s eye to ensure natural eye alignment and orientation. Stimuli appeared at 15° above the crabs’ visual horizon.

Experiment 1 contained three treatments, which allowed us to identify how crabs integrate signals from two simultaneously approaching predators. All stimuli were identical in contrast (− 100% Weber’s contrast). Treatment one was a single stimulus (Single, Fig. 1C). Treatment two contained two identical stimuli approaching simultaneously with an angular separation of 90° (Paired 90, Fig. 1C). Treatment three contained two identical stimuli approaching simultaneously with an angular separation of 180° (Paired 180, Fig. 1C). The crabs’ escape directions were analysed to see whether they used selective or divided attention. For instance, if a crab selectively attends to only one of the Paired 90 stimuli, this would result in the crab running directly away from that predator^35^, whereas divided attention will result in the crab integrating the signal from both predators and running in a direction that maximises the distance between both predator stimuli. In the Paired 180 treatment the two stimuli impose directly contradicting information on the neural system and selective attention would result in the crab running away from one predator but towards the other whereas divided attention would result in the crab running in a direction that successfully evades both. Therefore, the escape response to the Paired 180 may reflect a different mechanism compared to the Paired 90.

Experiment 2 contained four treatments: single low contrast (LC), single high contrast (HC), Paired 90 and Paired 180. Low and high contrast for the single treatments refer to a single looming stimulus where its’ pixel value was altered to produce contrast values (Weber’s contrast) of − 40% and − 100% with a constant background of 50% grey. Paired stimuli (Paired 90 and Paired 180) contained the simultaneous approach of a low contrast stimulus (− 40%) and a high contrast stimulus (− 100%) that differed in angular separation as described above. Changing the contrast of the stimuli was intended to modify the threat certainty level, with lower contrast hypothesised to elicit fewer and/or later responses (Preliminary results).

The order of presentation was randomized using a Latin square design, with crabs as columns and treatments as rows. This allows the randomization of stimulus order whilst ensuring that each crab sees every stimulus once and stimulus order was balanced across crabs^53^. Different Latin squares were used for experiments 1 and 2.

#### Response measures

Video footage was converted from MTS to AVI format using FFmpeg (ffmpeg.org). The crabs’ behaviour was then digitized using custom-made digitization software in MATLAB^54^. Using this software, the horizontal plane (x and y) position of each crab and its body orientation (angle in the horizontal plane with respect to the stimulus) was extracted, and all behaviours were recorded relative to the calculated ‘time to collision’ of the stimulus. Time to collision thus describes the difference between a behavioural observation and the time left before the theoretical collision of the virtual stimulus with the crab.

The initiation of the escape run was visually identified from video playback (a sample video of a crab responding to a looming stimulus is provided in the “Supplementary Material S1”). ‘Running’ was marked when all the legs of the crab moved rapidly at least one step in one direction. Escape direction (the direction in which the crabs ran relative to the stimulus), was manually digitized. The data was then imported into the open-source software R (R Core, 2017) for statistical analysis. For experiment 1, we tested for an effect of predator treatment (Single, Paired 90 or Paired 180) on the escape probability, timing, and escape direction (details in “Statistics” section below). In Experiment 2, we tested for an interaction of contrast and predator treatment (LC, HC, Paired 90 and Paired 180) on escape probability and timing (details in “Statistics” section below). A detailed analysis of the effect of stimulus contrast on escape direction will be presented in a separate paper.

#### Statistics

##### Response timing and probability

Linear Mixed Effects Models (LME) fitted with maximum likelihood and Generalised Linear Mixed Models (GLMMs) were used to test for an effect of treatment on stimulus time to collision and response probability respectively, using the R package ‘lme4’^55^. Time to collision was square root transformed to improve the distribution of residuals. We also tested for effects of carapace size, the crabs’ sex, and the order of presentations. Treatment and sex were treated as categorical variables while carapace size and order of presentation was treated as a continuous variable. A random intercept term of individual nested within group was included in all models to account for repeated measures on the same individuals and to account for effects associated with measuring groups of crabs at different times. Significance (p < 0.05) was determined by comparing the fit of each model to a reduced model and all p-values presented were estimated by comparing the fit of each model against a final model (using Likelihood Ratio Tests) that only contained significant terms. The assumptions of the models were checked by exploring the distribution of the residuals (using Q–Q plots) and examining plots of the standardized residuals against the fitted values for each model. Post-hoc tests were conducted to test for a difference between levels of our treatment factor by comparing the estimated marginal means from the model output using the R package ‘emmeans’^56^. P-values were adjusted for multiple comparisons using the Tukey method. This same package was used to extract the estimated marginal means and standard errors from the models to be used for plotting. The means and standard errors were back transformed for figures so that the values could be interpreted at an intuitive scale. Experiments 1 and 2 were analysed independently.

##### Circular statistics

In Experiment 1, where all stimuli had equal contrast, we found no difference in response probability and response timing as a function of treatment (see “Results”). We, therefore used circular statistics to detect any difference in mean running direction between predator treatment (Single, Paired 90 and Paired 180).

We looked for differences in running direction as a function of treatment and for the factors that correlate the running direction using circular mixed-effect models from the statistics package ‘bpnreg’^57^. In the experimental design, Single predator stimuli were randomly assigned to any one of the four computer monitors, and Paired predator stimuli were randomly assigned to any two of the four computer monitors. For statistical analysis, we therefore expressed all angles relative to the approach direction of the stimuli. In the Paired 90 treatment the data were rotated such that one of the stimuli approached from 0° and one from 270° and in the Paired 180 treatment one approached from 0° and one from 180° (Fig. 1C). Responses to the paired predator treatments showed a bimodal distribution and were therefore rotationally transformed to a unimodal distribution for comparison with the responses to the single predator treatment that was transformed analogously (“Supplementary Material S1”, Fig. S1). This was done by multiplying the raw angles by four (Single verse Paired 90) or two (Single verses Paired 180) and then taking their modulus with respect to 360°.

The bpnreg package takes a Bayesian approach to circular statistics and estimates model parameters with Markov chain Monte Carlo (MCMC) samplers (see^58^). All models contained running direction (degrees) as the response variable and crab identity as a random effect to account for multiple measures on the same individual. We then compared an intercept only model to a model that contained predator treatment to test whether treatment had a significant effect on the mean running direction. Models were compared using the deviance information criterion (DIC and DIC_alt_) and the Watanabe-Akaike information criterion (WAIC_1_ and WAIC_2_). In addition, a significant difference between treatment levels was determined by whether the 95% highest posterior density uncertainty intervals (HPD UI) for the lower bound (LB) and upper bound (UB) overlapped (no difference). For all models we ran 10,000 iterations with a burn-in period of 1000 iterations and a lag of three (i.e., keeping every third iteration). Trace plots were inspected to determine if the models had converged.

We tested for an effect of body orientation on running direction. For this purpose, we converted body orientation vectors into a categorical variable (90° bins) designed to capture when the predator stimuli approached the crabs’ lateral axis and/or the crabs’ anterior/posterior axis. We also tested for an effect of lateralization on escape responses in male crabs by marking the position of the large claw relative to stimulus approach direction. There was no effect of claw orientation (Supplementary Material Table S1). Therefore, we treated the escape directions to the Paired 180 treatment as axial data when testing for the effect of body orientation and transformed the data to a unimodal distribution for circular statistics.

### Electrophysiology

#### Apparatus and experimental procedure

A fiddler crab was removed from the holding facility and its carapace was glued with Loctite Superglue (ethyl cyanoacrylate) to a vertical metal rod. The rod with the crab was positioned in the centre of two LCD monitors (1920 × 1200 pixels at 60 Hz, Dell U2412M) positioned at right angles to each other (Fig. 1B). Both eyes were stabilized in an approximately natural position (~ 90° from the horizontal line) by gluing (Loctite Superglue) the eyestalks to a wooden post (Fig. 1B).

The animals then were anaesthetised by inserting them into a 3:1 mixture of crushed ice and seawater. To access to the LG neurons, a small incision was made using a sharp razor blade and removing a small piece of cuticle from the top of the eyestalk without causing damage to the ommatidial area. The crab was returned to room temperature by placing the lower part of their body in seawater. The crabs remained in seawater for the entire duration of the experiment thereafter. Microelectrodes of borosilicate glass tubing (World Precision Instruments, Inc; 1.2 mm outer diameter, 0.68 mm inner diameter) were pulled on a laser-based micropipette puller (P-2000, Sutter Instrument, Novato, CA, USA) yielding tip resistances of 60–100 MΩ when filled with 1 M potassium chloride. The electrode was then inserted into the optic lobe through the small incision. A shielded silver/silver chloride pellet was placed inside the seawater bath containing the crab and served as the reference electrode. Membrane potentials were recorded via an amplifier (Getting Model 5A, Getting Instruments, San Diego, CA, USA), connected to a computer via a 16-bit data acquisition card (USB-6353, National Instruments, Austin, TX, USA) sampling at 10 kHz.

The two monitors were mounted on a rotating platform and the crabs were positioned 27 cm from the screens on the centre of rotation. The back of the monitors and cables were covered with aluminium foil to reduce electrical noise. The entire setup was housed inside a Faraday cage.

Once penetration of the neuron was identified through the change in membrane potential, the cell type was identified by moving a hand around the animal and by delivering a flash of light^41^. The monitors were rotated around the crab to cover the receptive field of a neuron as estimated by the hand movements. The Faraday cage was completely covered with black block-out blinds to block all outside visual stimulation. As suggested by previous studies, the animal was left undisturbed for at least 3 min before the start of stimulation^42,43,59^. The inter-trial interval was set to 3 min.

#### Identification of neurons

To determine the identity of each neuron, we used characteristics that were previously identified by Medan et al.^41^ such as receptive field size, responses to squares moving horizontally or vertically, spontaneous activity, response to a light pulse, responses to a looming stimulus, and responses to a mild mechanical stimulation applied with a paintbrush to one of the animal’s legs. In total 17 MLG1 (12 from the left eye and 5 from the right eye) and 10 MLG2 neurons (4 from the left eye and 6 from the right eye) were recorded from, and their outputs analysed. Given the difficulty of intracellular recordings, experiments were repeated for 11 MLG1 and three MLG2 neurons, however, our statistical analysis (see “Statistics”) accounts for these repeated measures.

To test for the location of the receptive field and the directional selectivity of the cells, we measured the cells’ responses to a single black square (subtending 20° × 20° from the position of the crab) moving at a speed of 270 mm/s across both screens. The starting positions of the stimuli and the direction of movement was randomised along horizontal (left-to-right, right-to-left), and vertical (up-to-down, down-to-up) directions. Stimulation of MLG1 neurons resulted in differences in the spike frequency reflecting local inhomogeneity in the receptive field (“Supplementary Material S1”, Fig. S2A), similar to what was found in previous studies^41,42,45^. This inhomogeneity in the receptive field led to different response strength to identical stimuli presented in different locations in Experiment 1. We therefore categorized the single stimuli as ‘Primary’ or ‘Secondary’ based on their location in the receptive field (i.e., a Primary stimulus was closer to the hot spot of receptive field and produced a bigger response than a Secondary one). Unlike MLG1 neurons, MLG2 neurons have a more uniform looming sensitivity across the horizontal range of their visual field^42^ (see also Supplementary Fig. S2B for an example of MLG2 receptive field). Therefore, for MLG2 neurons, there was no way to separate the two single stimuli based on their response strength.

#### Stimuli and treatments

Similar to the behavioural experiments described above, monitors were used to display computer-generated looming stimuli. Looming stimuli simulated dark disks of 30 mm in diameter on a 50% grey background, approaching with constant velocity of 90 mm/s on a direct collision course towards the animal, starting from a distance of 1000 mm. To crabs these stimuli thus expanded from an object with a diameter subtending 1.7° until it filled the monitor.

In Experiment 1, we compared the response of the MLG1 and MLG2 neurons to single or paired looming stimuli with identical contrast. Experiments contained three treatments: single in the primary location (Primary), single in the secondary location (Secondary), and paired stimuli (Paired). The paired treatment always contained one stimulus in the primary and one stimulus in the secondary position of the receptive field. Since for MLG2 neurons there was no difference in response strength between the primary and secondary location, those stimuli were combined for analysis (Single). For both types of neurons, MLG1 or MLG2, the stimuli in the paired treatment were separated by 90° in the horizontal plane due to the positioning of the computer monitors.

In Experiment 2, we compared the responses of MLG1 and MLG2 neurons to single or paired looming stimuli that differed in contrast (− 100% and − 40% Weber contrast). The contrast manipulation used here was the same as was used in the behavioural experiment, but for the paired stimuli, only a 90° separation was included as only two computer monitors were used. The location, primary or secondary, of the low and high contrast stimulus in the receptive field of MLG1 neurons were balanced across neurons (i.e. the number of MLG1 neurons that have seen HC/LC in the primary location is equal to the number of MLG1 neurons that have seen the same stimulus in the secondary location). For the MLG2 neurons, the location of the HC and LC stimulus on the screen were randomised.

The order of stimulus presentation was randomized using a Latin square design, allowing randomization of stimulus order whilst ensuring that each neuron sees every stimulus once and that stimulus order is balanced across each type of neuron^53^. Different Latin squares were used for experiments 1 and 2. The computer monitors were calibrated for linearity in radiance as described above (see “Stimuli and treatments in behaviour” section).

#### Response measures

Each individual recording was convolved with a temporal Gaussian window with Full Width at Half Maximum of 200 ms. The instantaneous firing rate was estimated by normalizing the resulting waveform such that its integral was equal to the total number of spikes recorded over the entire trial^60^. The beginning of the response was determined via an inclusion criterion that the instantaneous firing rate should be above a threshold of 2 standard deviations of the spontaneous activity (i.e. unstimulated) for each cell.

#### Statistics

Time of response, peak spike rate and average spike rate were analysed using a linear mixed model analysis (LME, MATLAB R2019a). In all experiments, neurons reached their peak at the end of stimulation, therefore, we did not include time of peak in our analysis. Some response measures were logarithmically transformed to improve the distribution of residuals (see “Supplementary Material S1”, Tables S5 and Table S6). We tested for the effect of treatment and sex as categorical variables, and order of presentation as a continuous variable. The variance associated with individual crabs and individual neurons was taken into account by treating them as nested random factors. The final model, significance level, and the assumptions of the models were checked as described above for the behavioural analysis. Post-hoc tests were conducted to test all-pair comparisons for treatments and specify groups that differed. Post-hoc tests were performed using Tukey’s HSD with the Holm–Bonferroni adjustment using the R package ‘multcomp’^61^.

## Results

### Behaviour

#### Response timing and probability

**Figure 2:**
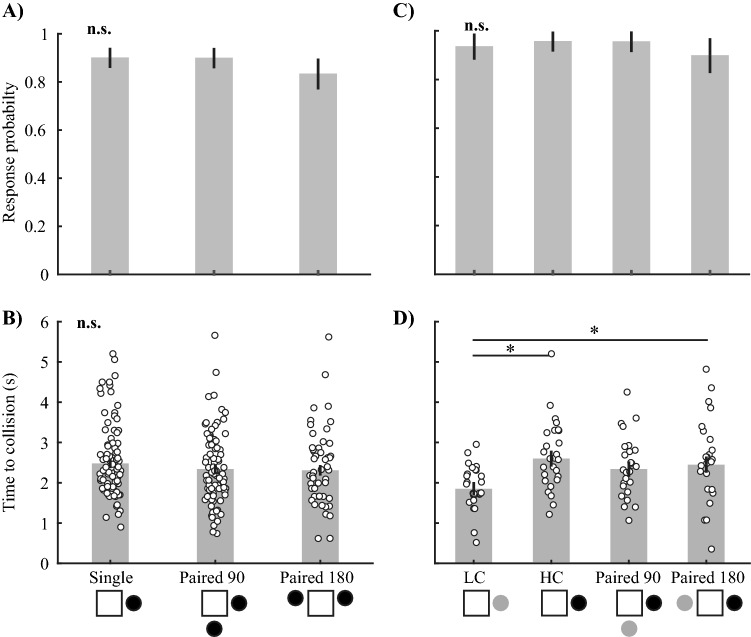
The probability and timing of the escape response to the approach of single and paired looming stimuli. There is no difference in (**A**) response probability and (**B**) response timing (time to collision) to single and paired stimuli of equal contrast (experiment 1). (**C**) Response probability also did not change in response to single or paired stimuli that varied in contrast (experiment 2). (**D**) A single low contrast (LC) stimulus elicited a later response than stimuli that contained a high contrast (HC) stimulus (regardless of whether they were paired with a LC stimulus or not). Diagrams below the x-axis show the number, contrast, and angular separation of looming stimuli. The square in the centre of each diagram represents the four computer monitors. Solid black circles represent looming stimuli of − 100% contrast and solid grey circles represent looming stimuli of − 40% contrast. All bars represent estimated marginal means (± SE) and white circles in (**B**) and (**D**) show each observation. The solid black lines and asterisk symbols (*) connect levels of treatments that were found to be significantly different (* 0.01 ≤ p < 0.05, n.s. = not significant). All statistical tests on response timing were performed on transformed data but were back transformed for display (see methods for details).

The crabs reliably responded to all simulated predator treatments with an average response probability of 75.4%. In Experiment 1, the crab’s response probability and timing did not differ between treatments, regardless of whether they were approached by one or two predators, or whether the paired predators were separated by 90 or 180° (probability: Fig. 2A, Supplementary Material Table S2, p = 0.32; timing of the escape run: Fig. 2B, Table S2, p = 0.37). We found a small but significant positive relationship between stimulus order and escape probability (Supplementary Material Table S2, p = 0.006) as well as escape timing (Supplementary Material Table S2, p = 0.001). This suggests the crabs became slightly sensitised to the stimuli. However, as the experimental design was fully balanced, this had no influence on the main treatment effect.

In Experiment 2, the probability of an escape run was also independent of whether the crabs faced a low or high contrast, or single or paired stimuli. (Fig. 2C, Supplementary Material Table S2, p = 0.71). This shows that both contrast levels reliably elicit responses, even when presented in isolation. However, there was a significant effect of predator treatment on response timing, where responses occurred significantly later to the low contrast stimulus (Fig. 2D, Supplementary Material Table S2, p = 0.004). We found no difference between any of the predator treatments that included the HC stimulus. Therefore, when crabs are exposed to two stimuli of differing contrast, the crabs’ response timing is similar to the timing in response to a single, high contrast stimulus.

#### Escape direction

**Figure 3:**
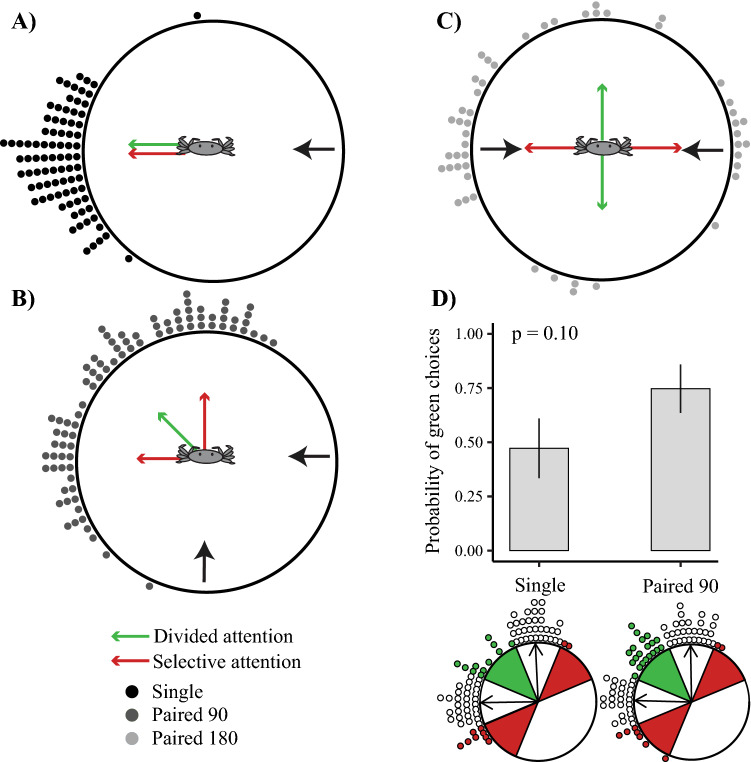
Escape directions in response to single or paired predators. Crabs were exposed to either (**A**) a single looming stimulus (Single, black circles), (**B**) paired looming stimuli that differed in their approach angle by 90° (medium-grey circles, Paired 90), or (**C**) paired looming stimuli that differed in their approach angle by 180° (light-grey circles, Paired 180). Black arrows show the predator approach direction, and the green and red arrows show the predicted running directions under a divided attention or selective attention mechanism respectively. (**D**) The divided attention hypothesis predicts a higher number of crabs to run into the green sector than to run into the combined red sectors. In contrast, the selective attention hypothesis predicts an equal distribution. To test this, we compared the running directions of crabs that faced a single predator with those that faced two predators. The probability of crabs running into the green or combined red sectors is not significantly different between the two treatments. The insets below the x-axis show circular diagrams of the binning protocol that captures the data points that align with divided attention mechanism (green circles) and those that do not (red circles). The white circles show the data that belongs to the central 45° of each distribution and therefore were not analysed. The black arrows in these insets show the mean running directions of the white points only. The p value shows the result of a generalized mixed effects model (GLMM, d.f. = 1, χ^2^ = 2.74, p = 0.10).

Irrespective of whether crabs use divided or selective attention, in response to a single stimulus, we expected the crabs’ escape directions to form a unimodal distribution opposite to the stimulus’ approach direction (Fig. 3A). When exposed to paired stimuli (Paired 90 or Paired 180), we expected different escape directions depending on whether the crabs use a selective or divided attention mechanism. Under the divided attention mechanism, the escape directions should maximise the distance between both stimuli, and therefore be directed at the circular average of the stimulus approach directions (Fig. 3B,C, green arrows). A prerequisite for selective attention is that the crabs only respond to one of the stimuli in isolation. Therefore, for selective attention, the prediction is bimodal distribution of escape directions, with the two peaks positioned opposite to the approach of the two stimuli (Fig. 3B,C, red arrows).

As expected, we found a unimodal directional response (Fig. 3A) for crabs running away from a single stimulus. When two stimuli approached simultaneously, in contrast, we found peaks opposite both stimuli (Fig. 3B,C). A selective attention mechanism requires suppression of the distracting stimulus in order to respond to another. Therefore, when the crabs are exposed to the paired stimuli, the resulting distribution should be bimodal and identical to the sum of two (rotated) unimodal distributions. To test if this is the case, we compared the circular distributions of the paired treatments to the single treatment by transforming the bimodal distributions found in the paired treatments to a unimodal distribution (see “Methods” for details, Fig. S1). The same treatment was applied to the Single distributions. All treatment groups were significantly directional post-transformation (Rayleigh’s test, p < 0.05) and the mean escape direction did not differ significantly between the single and paired treatments (Supplementary Material Table S3). However, there was significantly higher variation in the responses to the Paired 90 treatment (Rao Test, χ^2^ = 4.52, p < 0.05) and the Paired 180 treatment (Rao Test, χ^2^ = 28.6, p < 0.05).

In the Paired 90 condition, there appears to be a smaller third peak that differs from the two main response peaks and approximately align with the predictions from the divided attention mechanism (Fig. 3B, green arrow). This might suggest that some crabs run away from both stimuli simultaneously. Alternatively, this cluster of datapoints could also be the addition of the two tails of the two unimodal distributions. To test this, we looked for a bias in the crabs’ running direction away from the second stimulus. We combined and rotated the crabs’ responses to the single treatment such that one stimulus always approached from the 0° and the other from 270° (Fig. 1C), exactly matching the approach configuration of the Paired 90 treatment. We then compared this combined distribution with the distribution we found in response to the Paired 90 treatment. If the presence of an additional predator in the Paired 90 treatment had no effect, then we would expect it to be equally likely that the crabs escape into the green sector compared to the combined red sectors in both the Paired 90 treatment and combined single treatments (Fig. 3D, green or red 45° sectors in diagram). This did not seem to be the case. In the Paired 90 treatment, almost 75% of all escapes that ended up in either the red or green sectors ended up in the green sector. In contrast, the probability was almost exactly half for the Single treatment (47.4%), as expected. However, the difference between the two treatments just failed to be significant (GLMM, χ^2^ = 2.74, d.f. = 1, p = 0.098). Within the white sectors, we found no bias in the mean escape response direction of the two main peaks (Fig. 3D, black arrows/white circles) towards the green sector. This was true for both the Single and Paired 90 treatments. This result indicates that there was no reliable evidence for a general bias away from the second predator.

#### Effect of body orientation on escape direction

**Figure 4:**
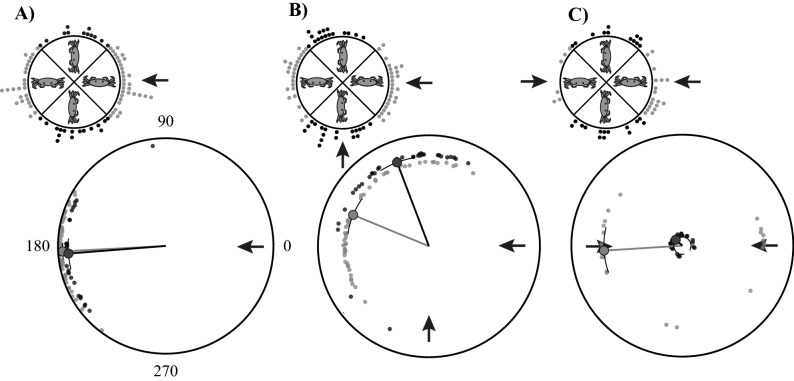
Escape direction of fiddler crabs as a function of their body orientation. (**A**) In response to a single stimulus, the crabs always ran directly away from the stimulus, irrespective of whether the stimulus approached the crabs laterally (grey) or along their posterior-anterior axis (black). (**B**) When the crabs faced the Paired 90 stimuli, they directed their escape run significantly more often away from the stimulus that aligned with their lateral body axes. Grey dots indicate runs where the 0° predator aligns with the crabs’ lateral axis and black dots runs where the predator at 270° aligns with the crabs’ lateral axis. (**C**) When confronted with the Paired 180 treatment, where the stimuli approach from either side (grey dots), there was no difference in mean escape direction, but when the stimuli aligned with the crabs’ posterior-anterior body axis (black dots), there was no directionality in the crabs escape response. The circular means and the uncertainty interval were calculated on transformed data (see Supplementary Material Table S4 for data transformation) but the data (small dots) are untransformed. Plot insets display the crabs’ initial body orientation relative to predator approach direction (black arrow). Each dot represents one observation, as either the orientation of the crab at the start of the escape (insets) or the escape direction (main plots). Data was binned in 90° angular sectors. Circular plots show the estimate of the circular mean (large dots) and 95% highest posterior density uncertainty intervals (UI, black curves). The length of the line segment that connects the circular mean to the plot centre indicates the mean resultant length (Rho).

In response to a single stimulus, the crabs always ran directly away from the stimulus, independent of their initial body orientation relative to the stimulus (Fig. 4A, Supplementary Material Table S4). However, in the Paired 90 treatment, we found a strong effect of body orientation on the mean running direction. The crabs were significantly more likely to run from the stimulus that approached the crabs’ lateral axis (Fig. 4B and Supplementary Material Table S4). The effect of body orientation on the mean escape direction was equally strong but different in the Paired 180 treatment. There was no difference in mean escape direction (Fig. 4C and Supplementary Material Table S4), but a significant difference in variation (Rao test, χ^2^ = 9.42, d.f. = 1, p < 0.05). When the crabs anterior/posterior axis was aligned with the approaching predators (Fig. 4C, black circles), their escape direction was no longer directed away from the stimuli.

### Neuronal responses

**Figure 5:**
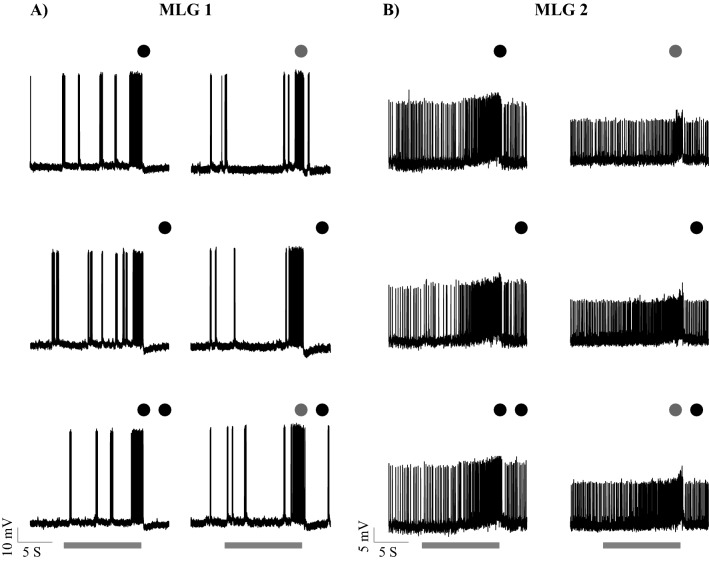
Example responses of (**A**) MLG1 and (**B**) MLG2 neurons to single and paired stimuli. Both neurons responded by gradually increasing their firing rate before the end of stimulation. The circles above the response traces show the type of stimulus used in each recording. A single circle represents a single stimulus and double circles represent paired stimuli. The colour of the circles indicated stimulus contrast (Webber contrast): − 100% (black) and − 40% (grey). The grey bar below the response trace shows the duration of stimulation.

To test the possible roles of MLG1 and MLG2 neurons in the selective attention of escape responses, we recorded intracellularly from these neurons and compared their responses to single and paired looming stimuli. Both cells respond to a looming stimulus by gradually increasing their rate of firing, which reached a peak near the time of predicted collision (Fig. 5).

If the neuron only selects one stimulus, the response to paired stimuli should resemble either of the two single stimuli when presented alone. However, if the neuron responds to both stimuli, we expect that the response to the paired stimuli would be a blend, such as their sum or average. We tested this by measuring the time of response, the peak of spike rate and the average spike rate during response.

#### MLG1 neurons

##### Experiment 1

**Figure 6:**
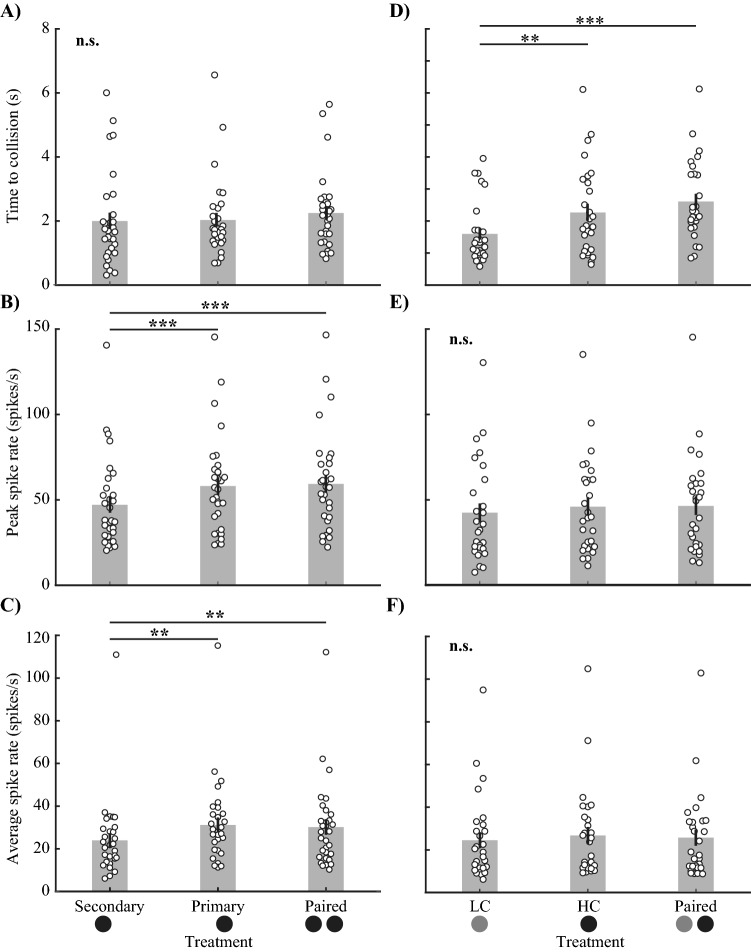
The firing parameters of MLG1 neurons in response to single and paired looming stimuli. (**A**–**C**) Experiment 1, stimuli of equal contrast; (**D**–**F**) Experiment 2, stimuli of different contrasts. When all stimuli were of equal contrast, there were no differences between (**A**) the time of the response regardless of the number of stimuli approached. (**B**) The peak spike rate and (**C**) the average spike rate were significantly lower for the single Secondary stimulus when compared with either the single Primary stimulus or Paired stimuli. However, the responses to Paired stimuli did not differ from those to the single Primary stimulus. When the stimuli differed in contrast, MLG1 neurons responded (**D**) significantly earlier to single high contrast (HC) or Paired stimuli compared to the single low contrast (LC) stimulus. However, the time of response was not significantly different between the single HC stimulus and the Paired stimuli. No differences were found for (**E**) peak spike rate or (**F**) average spike rate between any of the treatments. All bars represent means (± SE); white circles show individual measurements. The solid black line and asterisk symbols connect levels of treatments that were found to be significantly different through pair-wise testing (*0.01 < p < 0.05, **p < 0.01, ***p < 0.001, n.s. = not significant). All statistical tests were performed on transformed data but were back transformed for display.

MLG1 neurons responded to either of the single stimuli (Primary or Secondary) and the Paired stimuli at the same time (Fig. 6A and Supplementary Material Table S5). Both peak spike rate and the average spike rate were significantly higher during approaches of the single Primary and the Paired stimuli compared with the single Secondary stimulus (Fig. 6B,C and Supplementary Material Table S5). However, these response measures were not significantly different between the single Primary stimulus and the Paired stimuli (Fig. 6B,C, Supplementary Material Table S5).

##### Experiment 2

MLG1 neurons responded significantly earlier to the single HC stimulus and the Paired stimuli compared with the single LC stimulus (Fig. 6D, Supplementary Material Table S5, p < 0.001), but there was no difference between the Paired stimuli and the single HC stimulus (Fig. 6D, Supplementary Material Table S5). Peak and average spike rate did not differ between any of the treatments (Fig. 6E,F, Supplementary Material Table S5).

#### MLG2 neurons

##### Experiment 1

**Figure 7:**
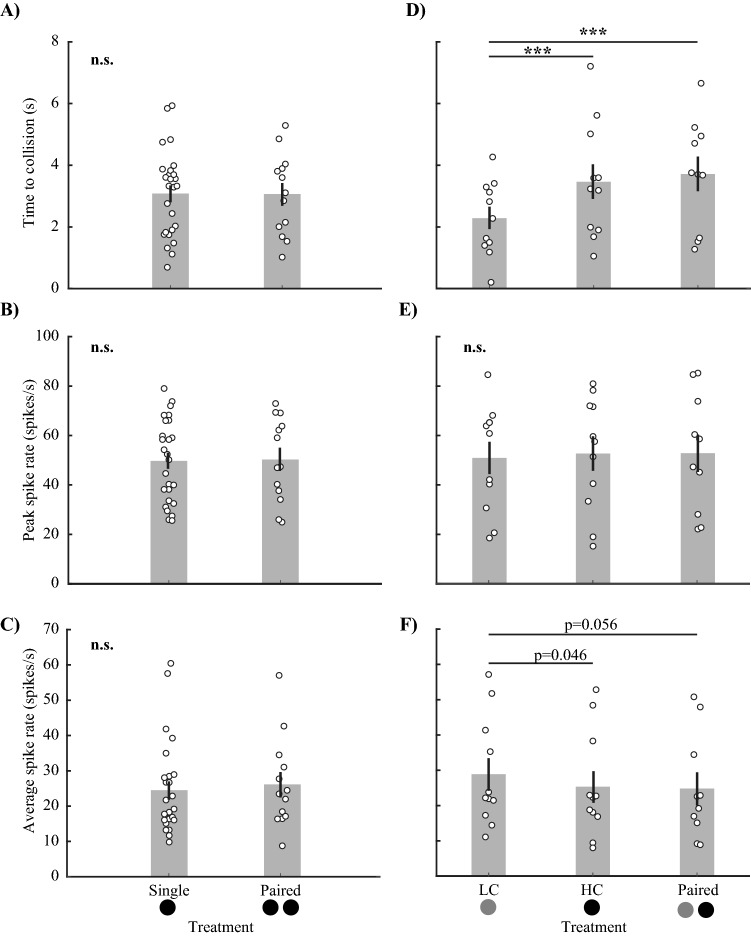
The firing parameters of MLG2 neurons in response to single and paired looming stimuli. (**A**–**C**) Experiment 1, stimuli of equal contrast; (**D**–**F**) Experiment 2, stimuli of different contrasts. When all stimuli had the same contrast, there were no differences in (**A**) the time of response, (**B**) peak spike rate, and (**C**) average spike rate, regardless of the number of stimuli that approached. (**D**) When the stimuli had unequal contrasts, MLG2 neurons responded significantly earlier to the single high contrast (HC) or Paired stimuli compared to the single low contrast (LC) stimulus. However, the response timing to the single HC stimulus was similar to that of the Paired stimulus. (**E**) There were no significant differences for the peak spike rate between either the single or paired stimuli of varying contrasts. (**F**) Average spike rate during the response to the single LC stimuli was higher than to the single HC stimulus and the Paired stimulus. However, the result was just not significant when comparing single LC with Paired stimulus. All bars represent means (± SE) and white circles show individual observations. The solid black line and asterisk symbols connect levels of treatments that were found to be significantly different through pair-wise testing (*0.01 < p < 0.05, **p < 0.01, ***p < 0.001, n.s. = not significant). The statistical tests that were performed on transformed data were back transformed for display.

We found no significant differences in the time or strength of MLG2 responses between the Single stimuli and identical Paired stimuli (Fig. 7A–C and Supplementary Material Table S6).

##### Experiment 2

MLG2 neurons responded significantly earlier to the single HC stimulus and the Paired stimuli compared with the single LC stimulus (Fig. 7D, Supplementary Material Table S6, p < 0.001) but again there was no difference between the Paired stimuli and the single HC stimulus (Fig. 7D, Supplementary Material Table S6). Peak spike rate did not differ between any of the treatments (Fig. 7E, Supplementary Material Table S6). However, the average spike rate during the response to the single LC stimuli was higher than both the single HC stimulus and the Paired stimuli but these differences were borderline (Fig. 7F, Supplementary Material Table S6, paired wise comparison p = 0.046 and p = 0.056 respectively). Given that the peak spike rates for all stimuli were similar, the average spike rate of MLG2 neurons to LC stimulus is expected to be higher because the neural activity changed from spontaneous activity rate to a same peak rate as the other stimuli in a shorter time. There was no difference between single HC and the Paired stimuli (Fig. 7 F, Supplementary Material Table S6, paired wise comparison p = 0.87).

## Discussion

Our study investigates the role of selective attention in the decision-making process of fiddler crabs when confronted with one or two simultaneously approaching predators. We compared the behavioural and neural responses of crabs presented with visual stimuli simulating approaching objects and showed that the crabs, and their MLG neurons, almost exclusively respond to only one of two simultaneous looming stimuli. When faced with two stimuli of different contrast, the MLG neurons and the crabs were much more likely to respond to the stimulus with the higher contrast. However, despite almost exclusively responding to only one stimulus, there is behavioural evidence that the crabs do not completely ignore the signal of one of the second stimulus. When approached by two identical predators from opposite sides, the crabs failed to differentiate between the predators and escape responses show less clear directionality. When the predators were separated by 90°, the crabs apparently biased their escape run slightly away from the second predator, but this bias was just below significance in statistical tests.

### Selective attention in fiddler crab escape

When faced with two simulated predator stimuli, the crabs almost completely ignored one of them. The addition of a second stimulus did not even lead to a change in their probability of response or response timing (Fig. 2). The direction of the crabs’ escape run clearly indicated that, in most cases, they ran directly away from one or the other stimulus rather than in a direction that maximizes the distance from both. This was even the case if that meant running directly towards the second stimulus in order to escape the first (Fig. 3C, Paired 180 stimulus). These results are mirrored in our recordings from MLG1 and MLG2 neurons. The timing or strength of the response of these neurons to stimuli of equal contrast did not differ between paired stimuli and the stronger of the two single stimuli (Figs. 6A–C and 7A–C). The combination of our behavioural and neural results clearly indicate that the crabs do not sum or average the signal from individual stimuli, but rather use a selective attention mechanism to suppress the response to one stimulus.

Despite strong evidence for selective attention, there is behavioural evidence that the crabs do not completely ignore the second stimulus. In the Paired 180 treatment, when the two stimuli approached the crabs from either the front or back (Fig. 4C), the crabs showed no significant directionality in their escape direction. This was not the case when a single stimulus approached the crabs from the front or back and in these experiments the crabs always rotated to escape in the direction directly away from the incoming predator (Fig. 4A).

There is also an effect of the second predator in the Paired 90 treatment. Between the two major peaks, which point directly away from either of the two stimuli, we found an additional, small peak, which is consistent with the crabs maximising the distance between both predators (Fig. 3B). However, our analysis could not statistically separate (p = 0.098) whether the escape trajectories that constitute this third peak result from the presence of two predators, or whether the peak is simply the addition of the two tails of the distributions centred on the main peaks. The crabs were clearly influenced by the presence of the second predator, as the distribution of escape directions included significantly more variation for treatments with two stimuli rather than one stimulus (Fig. 3). A potential approach for future experiments would be to increase the separation angle between the two predator stimuli to reduce the overlap between the two main escape response distributions. Regardless, our data provides evidence that the crabs do not totally ignore the signal of the second stimulus, but they do not seem to be able to respond in a way that effectively integrates both signals.

How do crabs decide which stimulus to escape from? When there is a difference in the contrast of the two simultaneously approaching stimuli (Experiment 2), the crabs responded only to the stronger stimulus and ignored the weaker stimulus. This finding is consistent with our previous observations of crabs in experiments conducted in the natural environment^14^. In these field experiments the crabs only responded to the more threatening dummy predator, which approached on a direct collision course, and ignored the less threatening predator which approach on a near-miss trajectory.

The results of our neural analysis mirror the behavioural results. When both stimuli were equal in contrast, the responses of the MLG1 neurons were consistent with what we would expect if they were only responding to the stimulus that was closer to the hotspot of their receptive field and therefore produced stronger responses. These results suggest that MLG1 neurons only process the stronger input signal and ignore the other one. Future experiments should account for possible effects of neural saturation, using paired stimuli of lower contrast or approaching at different velocities. However, it is unlikely that the neurons saturated in our experiments as all neurons reached their peak firing rate only at the very end of stimulation.

Both MLG1 and MLG2 neurons responded to single stimuli of both low and high contrast. However, when the stimuli were paired, the MLG neurons appeared to only respond to the high contrast stimulus and suppressed the response to the low contrast stimulus. In MLG1 neurons, which have non-uniform receptive fields, this result holds irrespective of the location in the receptive field at which the high contrast stimulus was presented (Primary or Secondary). It is not clear at this point whether this is solely driven by the stimulus contrast, or whether there is an interaction between stimulus contrast with the local strength of the receptive field at which the stimulus is presented. We only tested one level of contrast for the low contrast treatment. It is therefore possible that, if the contrast difference between the stimuli were smaller, the low contrast stimulus may have produced a higher response when presented in the primary location of the neurons’ receptive field compared to the high contrast stimulus positioned in the secondary location and therefore ‘winning’ the neuron’s selection. Future experiments are required to investigate this possibility in more detail.

In addition to the stimulus strength, the behavioural experiments showed that body orientation also has a strong effect in determining the escape direction. We found a strong effect of the crabs’ initial body orientation on running direction in both paired treatments (Fig. 4). In the Paired 90 treatment with two equal stimuli, the crabs escaped primarily from the predator that approached most closely to the crabs’ lateral axis. Therefore, crabs have a strong bias to simply run-away from the predator in a direction that most closely aligns with its preferred, sideways, mode of running. Similarly, in the Paired 180 trials, when the stimuli approached from the crabs’ lateral axis, the escape trajectories were highly directional and directed away from one of the two predators and towards the other. In contrast, the crabs did not have a preferred escape direction when the Paired 180 stimuli approached from the front and the back.

The preferred running direction of the crabs is also reflected in the makeup of their neural system. The ensemble of MLG1 neurons is distributed across the lobula in a retinotopic mosaic with a large overlap in their receptive fields^44,45^. Previous studies revealed that the majority of MLG1 units (10 out of 16 units) are dedicated to processing information from the lateral visual field of each eye^45^, which lines up directly with the crab’s lateral axis. Given such a distribution of receptive fields, a population vector of the MLG1 ensemble could be a primary determinant of the escape direction of the crabs and therefore influence stimulus selection, since the lateral stimulus is likely positioned closer to the hotspot of more MLG1 neurons (rather than the stimulus approaching from the front or back). Medan et al.^45^ previously argued that the precision of changes in the escape direction of crabs relative to the stimulus suggests that it is most likely represented by a population vector code from the ensemble of MLG1 neurons rather than a “winner-take-all” type mechanism that is driven by the MLG1 neuron that generates the strongest response. Although the existence of such a population vector has not yet been conclusively determined, the combination of our behavioural and neural data supports this interpretation.

Our neural experiments did not control for the location of stimuli relative to the crabs’ body axis. However, our recordings were from immobilized animals, which is not ideal for investigating the correlation between the cognitive behaviours and the underlying neural activity^62,63^. Extracellular recordings from several MLG1 neurons in tethered crabs could provide the required evidence for a population vector.

Irrespective of whether the final escape direction is the results of a putative population vector or not, there are at least two layers of neurons involved in the crabs’ selective attention during escape decisions. One layer comprises the MLG neurons themselves, and the other is a downstream layer. It is not clear yet what position the MLG neurons occupy in this selective attention process. Whether they are the first node showing the effects of attentional mechanism in this pathway, or whether this process starts within upstream neurons. It is easy to see how the selection of contrast can be supported by the MLG neurons, but it is less obvious how the predictive selection we found in our fieldwork^14^ could be supported at this level. In the latter experiment, the crabs selected the stimulus that was going to become the more dangerous stimulus as it was moving closer to a direct collision course and ignored the near miss one that initially generated a higher retinal speed.

Support for attention-like neural processes in arthropods have been found in every neuropil, except the outermost layers of the optic lobes (see review by De Bivort and Van Swinderen^64^). Although the looming-sensitive pathway is not yet fully identified in crabs, this circuit is well characterized in *Drosophila*. Recent work by Klapoetke et al.^65^ suggests that the lobula plate/lobula columnar type II (LPLC2) cell is a presynaptic partner of the GF interneuron, spike in response to looming stimuli, and provides highly specific information about looming stimuli. The LPLC2 neurons, which receive input from directionally selective neurons and encode the motion of bright (T4) and dark (T5) edges, are being excited by motion outward from the centre of their receptive field and inhibited by inward motion^65^, thus conveying information about angular size in looming objects. Additionally, a second presynaptic partner of the GF interneuron, the type 4 lobula columnar (LC4) cell, is tuned to rapid expansion of objects, encoding information about the angular velocity of looming objects^66^. Given the similarities in visual processing across arthropods^67–69^, underpinned by the monophyly of the Pancrutsacea^70^, it is likely that the looming sensitive pathway in fiddler crabs is driven by interneurons with similar mechanisms. Like most small invertebrates, crabs heavily rely on cues of moving objects such as angular size, angular velocity, and expansion speed^28,32,33^ for their escape decisions. To what extent other stimulus characteristics play a part in the selection process remains to be seen. It is possible, or even likely, though, that their selective attention mechanism starts at such interneurons that calculate the critical information from the dynamics of looming and or moving objects. However, further investigations will be required to identify the role of interneurons presynaptic to MLGs in crabs and their role in selective attention.

### Body orientation and escape trajectory

Body orientation has been shown to be an important determinant of the escape trajectory in other animals (collembolan springtails (*Heteromurus nitidus*)^71^, mosquito pupa (*Culex pipiens*)^72^, fish (*Pagrus major* and *Pterophyllum eimekei*)^73,74^, lizards (*Psammodromus algirus*)^75^). This escape strategy is probably determined by functional constraints in the manoeuvrability of the prey animal. For example, when the lizard *P. algirus* is not oriented towards the stimulus, it runs directly away, however, when the lizard’s initial orientation faces the stimulus, they were equally likely to run towards it^75^. Changing direction may be too time-consuming when facing the predator and therefore the lizards may be attempting to undercut the predator and force it to alter its trajectory rapidly^75^. Fiddler crabs run quickest when moving sideways^49^ and therefore the speed of escape can be maximised by running from a predator that most closely aligns with their lateral axis. When the fiddler crab *Uca pugilator*, was positioned on a polystyrene foam ball, the animals continuously rotated about the vertical axis in order to keep the presented predatory stimulus as close to their lateral axis as possible^26^. In addition, the lateral part of the fiddler crab eye has the highest resolution and therefore, by aligning their body axis to maximise the speed of escape (Fig. 4 and Fig. S3), the crabs also keep the predator in the region of highest visual resolution, both in terms of their ommatidial sampling array and the centre of their MLG1 array.

### Selective attention from an ecological perspective

Recent studies have shown that well-developed attention-like processes can be found in arthropods with relatively simple nervous systems including insects^12,36,64,76–80^, spiders^81,82^, and crustaceans^14^. Evidence of selective attention in the motion sensitive neurons of arthropods have previously been reported by^80^. They compared the response of the centrifugal small target motion detector (STMD) neurons in dragonflies to single and paired stimuli and showed that this neuron competitively selects one target in the presence of distracters. However, our study is the first to report selective attention in a looming sensitive neuron. In contrast to our findings, previous recordings in the descending contralateral movement detector (DCMD) neurons of locusts, which are postsynaptic to the looming-sensitive neurons, showed that responses to simultaneous approaches were almost identical to responses evoked by approaches of a single stimulus coming from the average approach angle. This indicates a divided attentional mechanism in the neural system underlying locust escape responses^83^. The reason for such differences between similar neurons might not be surprising given the substantial differences between the lifestyles of these animals. However, which aspects of their ecology drive these differences remains to be determined.

Selective attention is critical for dragonflies hunting individuals within a swarm of prey^80^, bees choosing between flowers^77^, or female crickets selecting between possible mates^78^. Nonetheless, selective attention in the context of predator avoidance, as we have observed in fiddler crabs, might not be an ideal solution as the animal risks being captured by the ignored predator. However, neural computations are energetically costly and time consuming, and escape decisions require fast and decisive responses. Moreover, acquiring more information and performing additional neural computations does not necessarily result in better or more effective decisions^84^. For burrow-centred fiddler crabs^18^, responding to only the most threatening stimulus is a good-enough solution because, in most situations a successful escape only requires the crabs to run back to their burrow, regardless of the direction of attack. Only crabs that are stranded on the surface without a burrow must decide in which direction to run. Instead of making decisions that encapsulate all the available information, animals with a small brain and low computational power, such as fiddler crabs, may well benefit from making fast and energy efficient choices that work in most situations^85,86^.

## Conclusion

Here, we provided both behavioural and neural evidence for selective attention in the context of escape decisions. Our results indicate that fiddler crabs are most likely to run from the stimulus that generates the strongest signal in their MLG neurons when confronted with two simultaneous predatory stimuli. In situations where both stimuli represent the same level of threat, the crabs’ body orientation has a large impact on which stimulus they choose to escape from. Our data provides evidence that while the crabs do not totally suppress the signal of one of the approaching stimuli, they are not able to effectively integrate both signals. MLG neurons play an important role in the crabs’ selective attention process. Our neural and behavioural data suggest that the final escape direction is determined by a population vector downstream of the MLG1 neurons.

## Supplementary Information

Supplementary Information.

Supplementary Video 1.

## Data Availability

The datasets generated and/or analysed during the current study are not publicly available as it is partially required for a future publication but are available from the corresponding author on reasonable request.
