# Supplementary material for: Behavioural and neural responses of crabs show evidence for selective attention in predator avoidance
Source: Sci Rep. 2022 Jun 15;12:10022. doi: 10.1038/s41598-022-14113-0 (PMC9200765; doi:10.1038/s41598-022-14113-0)
Supplement: Supplementary file 1 — Supplementary Information. [file 41598_2022_14113_MOESM1_ESM.pdf]

Behavioural and neural responses of crabs show evidence for selective attention in predator avoidance

Zahra M. Bagheri[1,2*], Callum G. Donohue[1,2*], Julian C. Partridge [2], and Jan M. Hemmi[1,2]

Supplementary Material

Table S1. Summary of model fit from Bayesian circular mixed models. The models test for an effect of male claw position on running direction for all three treatments (Single, Paired 90, and Paired 180). For each treatment, an intercept only model was compared to a model containing crab orientation as a fixed effect. Lower values indicate better fits. N=number of crabs and n=number of observations.

Comparison	N (n)	Fixed effects	DIC	DIC_{alt}	$WAIC_1$	$WAIC_2$
**Single**	22 (38)	Intercept only	25.22	26.65	25.36	25.53
		Claw position	32.11	33.58	30.92	33.17
**Paired 90**	25 (43)	Intercept only	114.08	119.79	115.07	115.07
		Claw position	115.20	135.00	118.11	119.83
**Paired 180**	21 (26)	Intercept only	97.56	99.60	97.96	98.05
		Claw position	104.60	117.22	107.38	110.16

Table S2. Result of a generalized linear mixed effect model analysis (GLMEs) on response probability and a linear mixed effect model analysis (LMEs) on response timing. Fixed effects found to be significant, and therefore included in the final model are shown in bold. (1|group/crab) was incorporated as random effect into the models. Time to collision was square root transformed to improve the distribution of residuals. The P values resulted from pairwise comparison for time to collision of Experiment 2 is as follow: LC –HC (p = 0.01); LC – P 90 (p = 0.10); LC – P 180 (p = 0.04). N=number of crabs and n=number of observations.

	Experiment	N (n)	Fixed effects	d.f.	Chi-squared	P - value
Probability	Experiment 1	50 (289)	Treatment	2	2.84	0.24
			Sex	1	0.26	0.61
			Carapace width	1	0.36	0.55
			**Stimulus order**	1	7.53	0.006
	Experiment 2	30 (118)	Treatment	3	1.38	0.71
			Sex	1	1.80	0.18
			Carapace width	1	2.07	0.15
			Stimulus order	1	0.01	0.92
Time to collision	Experiment 1	50 (118)	Treatment	2	1.95	0.38
			Sex	1	0.51	0.48
			Carapace width	1	0.01	0.92
			**Stimulus order**	1	10.16	0.001
	Experiment 2	30 (103)	**Treatment**	**3**	**12.90**	**0.005**
			Sex	1	0.65	0.42
			Carapace width	1	1.21	0.27
			Stimulus order	1	3.63	0.06

Table S3. Summary of model fit from Bayesian circular mixed models testing for an effect of predator treatment on crab escape direction. The table shows four metrics of model fit used to compare a model with predator treatment to an intercept only model. Lower values of all metrics indicate a better fit. The treatment fixed effect is a two-level factor for Single and Paired 90 predator treatments (row 1) or Single and Paired 180 treatments (row 2). The data in each comparison has been transformed to a unimodal distribution. N=number of crabs and n=number of observations. The $\theta = 4 \times \theta \ mod \ 360$ and θ= 2×θ mod 360 transformation was applied on the escape directions of Single vs Paired 90 and Single vs Paired 180 respectively.

Comparison	N (n)	Fixed effects	DIC	DIC_{alt}	$WAIC_1$	$WAIC_2$
Single vs Paired 90	50 (168)	Intercept only	580.52	584.53	581.66	581.68
		Treatment	580.36	584.29	581.60	581.69
Single vs Paired 180	50 (134)	Intercept only	362.34	396.76	367.28	366.67
		Treatment	349.15	357.78	350.75	350.87

Table S4. Summary of model fit from Bayesian circular mixed models. The models test for an effect of crab body orientation on running direction for all three treatments (Single, Paired 90, and Paired 180). For each treatment, an intercept only model was compared to a model containing crab orientation as a fixed effect. Lower values indicate better fits. Bolded values indicate that the orientation model is a better fit than the intercept only model for all four metrics. N=number of crabs and n=number of observations. The data for the Paired 180 treatment has been transformed to a unimodal distribution using θ= 2×θ mod 360 transformation.

**Predator type**	**N (n)**	**Fixed effects**	**DIC**	**DIC_{alt}**	**$WAIC_1$**	**$WAIC_2$**
Single	45 (84)	Intercept only	61.87	62.52	62.26	62.42
		Orientation	64.69	66.41	64.54	66.17
Paired 90	47 (84)	Intercept only	215.46	222.49	216.55	216.58
		**Orientation**	**194.78**	**199.96**	**195.90**	**196.19**
Paired 180	42 (50)	Intercept only	96.28	532.97	147.71	155.10
		Orientation	142.38	343.67	160.26	159.47

Table S5. The results from linear mixed effect model (LMEs) analyses for MLG1 neurons. (N=number of crabs, n=number of neurons). Fixed effects found to be significant are shown in bold. $(\mathbf{1}|\boldsymbol{Neuron}) + (\mathbf{1}|\boldsymbol{Crab})$ was incorporated as random effect into the models. Time of response is measured as time to the virtual collision. The response measures were log transformed to improve the distribution of residuals. The P values of paired wise comparisons are as follow:

+ HC – P (p=0.09); LC – P (p<0.001) ***; LC – HC (p<0.001) **,

++ primary - paired (p=0.67); secondary – paired (p <0.001) ***; primary - secondary (p<0.001) ***,

+++ primary - paired (p=0.56); secondary – paired (p <0.01) **; secondary - paired (p<0.01) **

	**Experiment**	**N (n)**	**Fixed effects (x_i)**	**d.f**	X^2	***P***
**Time of Response** [a]	Experiment 1	13 (17)	Intercept	91	-	-
			Treatment	2	4.72	0.09
			**Sex**	1	7.07	**<0.01**
			Stimulus order	1	3.34	0.07
	Experiment 2[+]	13 (16)	Intercept	81		-
			**Treatment**	2	24.7	**<0.001**
			Sex	1	3.15	0.07
			Stimulus order	1	1.78	0.18
**Peak spike rate**	Experiment 1[++]	13 (17)	Intercept	90	-	-
			**Treatment**	2	24.85	**<0.001**
			Sex	1	0.48	0.49
			Stimulus order	1	2.20	0.13
	Experiment 2	13 (16)	Intercept	83	-	-
			Treatment	2	2.94	0.23
			Sex	1	0.52	0.47
			Stimulus order	1	0.66	0.42
**Average spike rate**	Experiment 1 [+++]	13 (17)	Intercept	90		-
			**Treatment**	2	13.80	**<0.001**
			Sex	1	0.11	0.74
			Stimulus order	1	1.60	0.21
	Experiment 2	13 (16)	Intercept	83	-	-
			Treatment	2	2.02	0.36
			Sex	1	2.04	0.15
			Stimulus order	1	1.02	0.31

Table S6. The results from linear mixed effect model (LMEs) analyses for MLG2 neurons. (N=number of crabs, n=number of neurons). Fixed effects found to be significant are shown in bold. $(\mathbf{1}|\boldsymbol{Neuron}) + (\mathbf{1}|\boldsymbol{Crab})$ was incorporated as random effect into the models. Time of response is measured as time to the virtual collision. The peak spike rate for Experiment 1 was log transformed to improve the distribution of residuals. The P values of paired wise comparisons are as follow:

[+] paired wise comparison: HC - P (p=0.72); LC – P (p<0.001); LC - HC (p< 0.001),

[++] paired wise comparison: HC-P (p=0.87); LC-P (p=0.056); LC-HC (p=0.046)

	**Experiment**	**N (n)**	**Fixed effects (x_i)**	**d.f**	**X^2**	**P**
**Time of response** [a]	Experiment 1	10 (10)	Intercept	38	-	-
			Treatment	1	0.01	0.91
			Sex	1	0.08	0.78
			Stimulus order	1	2.06	0.15
	Experiment 2[+]	9 (9)	Intercept	29		-
			**Treatment**	2	16.97	**<0.001**
			Sex	1	1.40	0.24
			Stimulus order	1	0.006	0.94
**Peak spike rate**	Experiment 1	10 (10)	Intercept	38	-	-
			Treatment	1	0.07	0.78
			Sex	1	2.00	0.16
			Stimulus order	1	1.65	0.22
	Experiment 2	9 (9)	Intercept	31	-	-
			Treatment	2	0.65	0.72
			Sex	1	3.65	0.056
			Stimulus order	1	2.49	0.11
**Average spike rate**	Experiment 1	10 (10)	Intercept	37	-	-
			Treatment	1	2.22	0.13
			**Sex**	1	10.22	**<0.01**
			Stimulus order	1	0.39	0.53
	Experiment 2[++]	9 (9)	Intercept	28	-	-
			**Treatment**	2	6.84	**0.03**
			**Sex**	1	8.25	**<0.01**
			Stimulus order	1	1.00	0.32

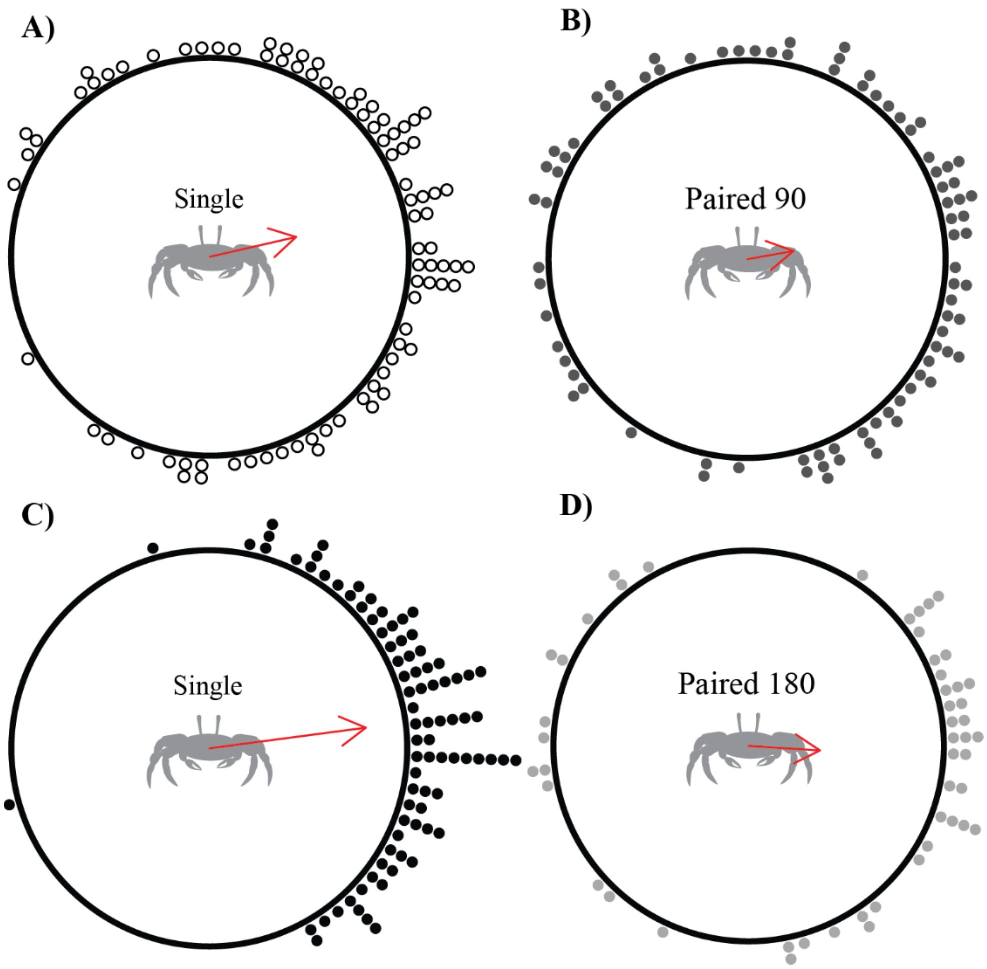

Figure S1. Transformed escape directions in response to single or paired predators. (A) The crabs escape directions to the Single stimulus (open circles) and (B) the escape directions to the Paired 90 treatment (dark-grey circles) were transformed to a unimodal distribution so that they could be statistically compared. This was done by multiplying the raw angles by four and taking their modulus with respect to 360°. (C) Similarly, the crabs escape directions to the Single stimulus (black circles) and (D) the escape directions to the Paired 180 treatment (light-grey circles) were also transformed to a unimodal distribution for comparison. This was done by multiplying the raw angles by two and taking their modulus with respect to 360°. The red arrow shows the mean escape direction and the length of the arrow shows the mean resultant length (rho).

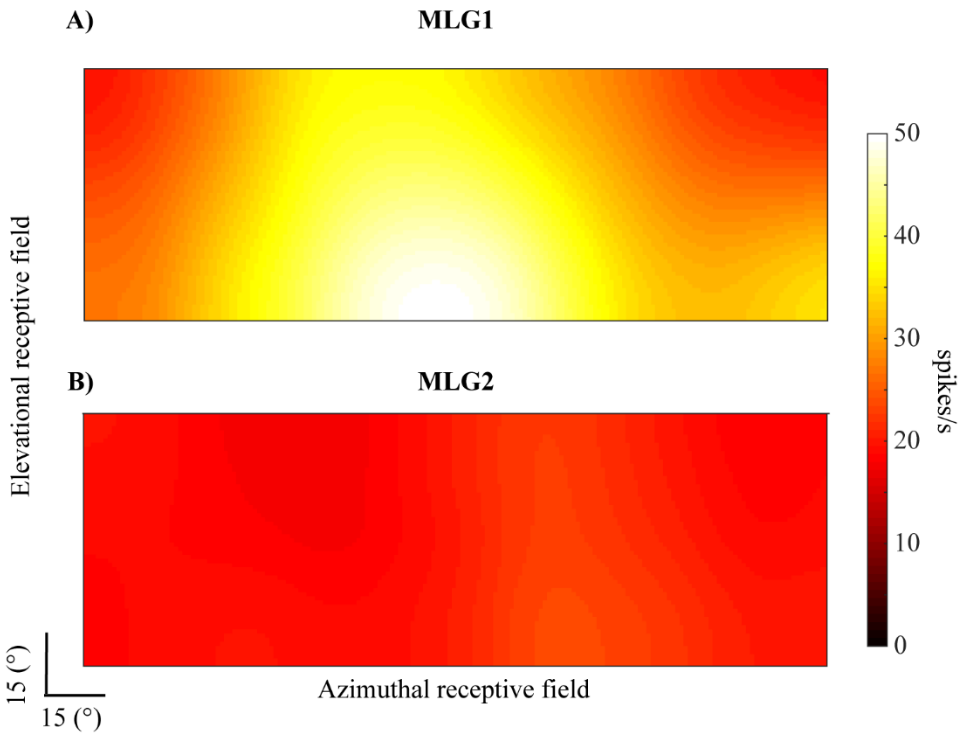

Figure S2. Examples of receptive fields of A) MLG1 and B) MLG2 neurons. The differences in the spike frequency of MLG1 neurons, reflected local inhomogeneity in their receptive field. We use this inhomogeneity in the receptive field to identify which of the two alternative identical stimuli in experiment 1 would result in a stronger neural response. Unlike MLG1, MLG2 neurons have a more uniform looming sensitivity across the azimuthal visual field.

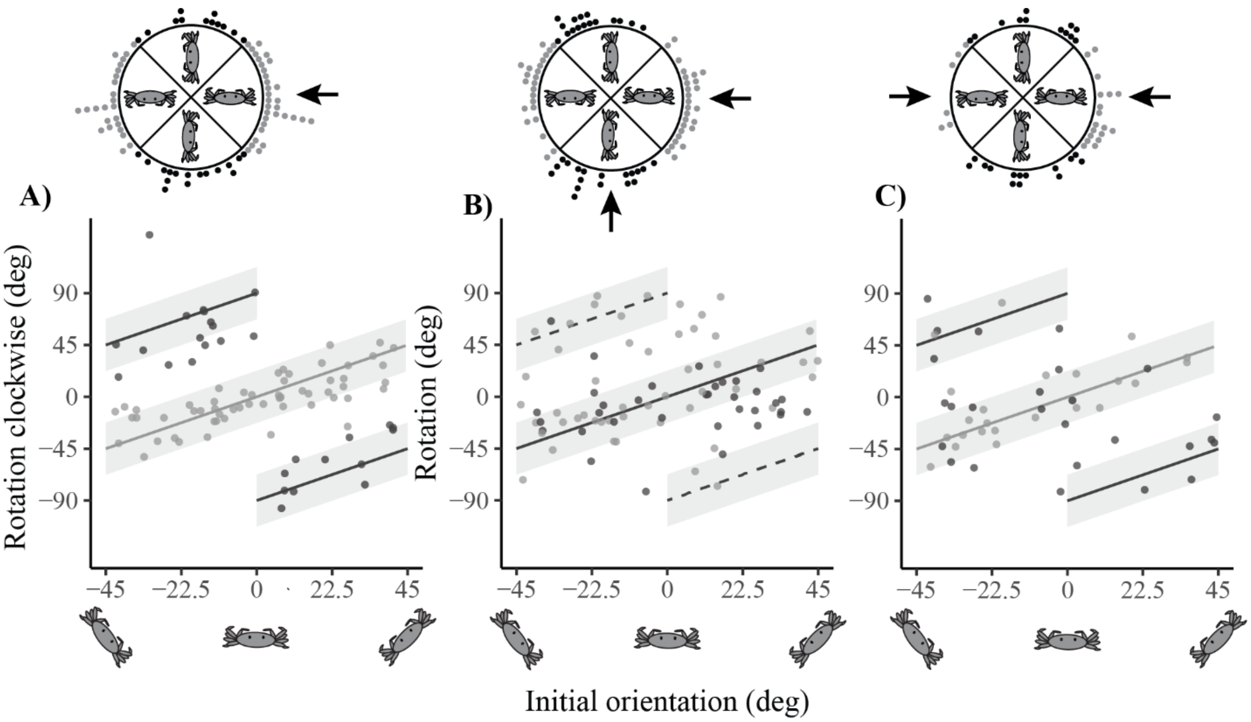

Figure S3. The correlation between initial body orientation and the crabs' rotation (clockwise is positive) to single and paired stimuli. Initial body orientation and rotations are relative to each of the four 90° circular sectors shown in the inset above the plots, so that an initial body orientation of 0 indicates that the crab is aligned with the centre of the sector. The lines are the expected rotations required to escape from the stimulus and the circles are the crabs' actual rotations. A) When presented with a single stimulus, crabs rotated the minimum amount required to escape, independent of whether the stimulus approached the crab laterally (grey) or anteriorly-posteriorly (black). B) When presented with the Paired 90 treatment, more crabs rotated less than 45 degrees to align with the predator closest in orientation to their lateral axis (solid line) with fewer crabs rotating to escape from the predator that approached closer to their anterior – posterior axis (dashed line). C) When the Paired 180 stimuli approached the crabs lateral body axis, the crabs rotated the amount required to align laterally and escape (grey), but the crabs seemed to rotate random amounts when the stimuli approached the crabs anterior/posterior axis (black lines). Points that fall within the grey shading represent crabs that successfully rotated to escape from the stimulus represent by the line (grey shading = ± 22.5°).

Supplementary Video. A fiddler crab responds to a looming stimulus in one of our experiments. At the beginning of stimulation, the fiddler crab (the left window), which is held on a treadmill, mostly walks whilst attempting to feed from the surface of the polystyrene foam ball. The treadmill is surrounded by four monitors and the looming stimulus (the right window) is presented on the right monitor. When the crab detects the stimulus, it ‘freezes’ (complete lack of movement of all limbs) followed by a very distinctive ‘run’ behaviour. Directly prior to the apparent collision of the stimulus with the crab, the crab ‘brace’ during which it tucks its legs and claws close to the body and fold its eyes into protective grooves in the carapace.
